# Feasibility of CBCT‐based dose with a patient‐specific stepwise HU‐to‐density curve to determine time of replanning

**DOI:** 10.1002/acm2.12127

**Published:** 2017-07-13

**Authors:** Shifeng Chen, Quynh Le, Yildirim Mutaf, Wei Lu, Elizabeth M. Nichols, Byong Yong Yi, Tish Leven, Karl L. Prado, Warren D. D'Souza

**Affiliations:** ^1^ Department of Radiation Oncology University of Maryland School of Medicine Baltimore MD USA; ^2^ Department of Radiation Oncology University of Maryland Medical Center Baltimore MD USA; ^3^ Department of Radiation Oncology Boston University School of Medicine Boston MA USA

**Keywords:** adaptive radiation therapy, CBCT‐based dose calculation, HU‐to‐density curve

## Abstract

**Purpose:**

(a) To investigate the accuracy of cone‐beam computed tomography (CBCT)–derived dose distributions relative to fanbeam–based simulation CT‐derived dose distributions; and (b) to study the feasibility of CBCT dosimetry for guiding the appropriateness of replanning.

**Methods and materials:**

Image data corresponding to 40 patients (10 head and neck [HN], 10 lung, 10 pancreas, 10 pelvis) who underwent radiation therapy were randomly selected. Each patient had both intensity‐modulated radiation therapy and volumetric‐modulated arc therapy plans; these 80 plans were subsequently recomputed on the CBCT images using a patient‐specific stepwise curve (Hounsfield units‐to‐density). Planning target volumes (PTVs; D98%, D95%, D2%), mean dose, and V95% were compared between simulation‐CT–derived treatment plans and CBCT‐based plans. Gamma analyses were performed using criterion of 3%/3 mm for three dose zones (>90%, 70%~90%, and 30%~70% of maximum dose). CBCT‐derived doses were then used to evaluate the appropriateness of replanning decisions in 12 additional HN patients whose plans were previously revised during radiation therapy because of anatomic changes; replanning in these cases was guided by the conventional observed source‐to‐skin‐distance change‐derived approach.

**Results:**

For all disease sites, the difference in PTV mean dose was 0.1% ± 1.1%, D2% was 0.7% ± 0.1%, D95% was 0.2% ± 1.1%, D98% was 0.2% ± 1.0%, and V95% was 0.3% ± 0.8%; For 3D dose comparison, 99.0% ± 1.9%, 97.6% ± 4.4%, and 95.3% ± 6.0% of points passed the 3%/3 mm criterion of gamma analysis in high‐, medium‐, and low‐dose zones, respectively. The CBCT images achieved comparable dose distributions. In the 12 previously replanned 12 HN patients, CBCT‐based dose predicted well changes in PTV D2% (Pearson linear correlation coefficient = 0.93; *P* < 0.001). If 3% of change is used as the replanning criteria, 7/12 patients could avoid replanning.

**Conclusions:**

CBCT‐based dose calculations produced accuracy comparable to that of simulation CT. CBCT‐based dosimetry can guide the decision to replan during the course of treatment.

## 1 INTRODUCTION

The need for adaptive radiotherapy has been demonstrated by many investigators.^1^, ^2^, ^3^ New plans are adapted throughout the weeks‐long course of fractionated radiotherapy to account for patient geometry changes resulting from weight loss, organ deformation, tumor shrinkage, and other causes. New adaptive plans may also be needed if the immobilization device needs to be adjusted or remade for variety of reasons. For some patients receiving intensity‐modulated radiotherapy (IMRT) or volumetric‐modulated arc therapy (VMAT), the significant benefit of replanning has been demonstrated.^4^ The frequency of replanning in patients with head and neck cancer was reported to be 32%–70%, depending on criteria.^5^ It is challenging, however, to decide on the appropriate time for replanning. Several investigators have looked for indicators to predict substantial dosimetric change. Although correlations between several parameters (such as weight loss, skin separation, and others) and dose change to target or organ at risk (OAR) were observed,^4^, ^5^, ^6^ no single parameter can be reliably used to decide the time of replanning for patients with head and neck cancer.^4^ Therefore, decisions on replanning are frequently based on the practical experience of clinicians.

The main challenge in initiating the replanning process is a lack of tools for estimation of dosimetric changes for targets and OARs. Onboard kV cone‐beam CT (CBCT) is now widely available, and CBCT‐based dose calculation makes it possible to evaluate dosimetric change during the course of treatment. Although kV CBCT technology is mainly used to set up patients and localize anatomy, its potential for use in dose calculation has been recognized and reported.^7^, ^8^, ^9^, ^10^ Dose calculation accuracy using CBCT images has been evaluated by investigators.^8^, ^9^, ^11^, ^12^, ^13^ The main source of dosimetric error stemming from CBCT‐based dose calculations (relative to fan‐beam‐based CT simulation) comes from the uncertainty in Hounsfield unit (HU)‐to‐electron density conversion of CBCT images. As a more direct approach, phantom‐based calibration of the HU‐to‐electron density curve was investigated.^7^, ^8^, ^9^, ^13^ Unlike fan‐beam CT, kV CBCT suffers from scatter, which results in greater HU uncertainty.^13^, ^14^, ^15^, ^16^ Because CBCT HUs vary with disease site, scanning mode, scanning range, and other factors,^13^ multiple calibration curves may be required. Moreover, HUs are dependent on patient size,^17^, ^18^ making this method even more challenging. An alternative method that relies on mapping of electron density values from planning CT to CBCT images was introduced.^8^, ^19^, ^20^, ^21^ Because of the complexity of these methods, CBCT‐based dose is not commonly used in routine clinical practice.

A recently developed treatment planning system RayStation V5.0 (RaySearch Laboratories; Stockholm, Sweden) provides CBCT‐based dose calculation using a patient‐specific stepwise HU‐to‐density curve (i.e., patient CBCT HUs were converted to only six classes of materials: air, lung, adipose, tissue, cartilage/bone, and other high‐density material). The method is similar to density override in its assignment of six classes of materials. Most modern treatment planning systems provide a density override function, so that this method could be used widely in clinical practice. The purpose of this study was to (a) investigate the accuracy of CBCT‐based dose calculations in the RayStation treatment planning system, and (b) study the feasibility of using CBCT‐based dose to select the appropriate treatment replanning time. In this study, dose calculation accuracy was assessed using 80 IMRT/VMAT plans for four anatomic sites: head and neck (HN), lung, pancreas, and prostate. The appropriateness of replanning decisions was evaluated with data from 12 rescanned patients with head and neck cancer.

## 2 METHODS AND MATERIALS

### 2.A Patient data

Image data from 40 patients who underwent radiation therapy at our institution were randomly selected for this institutional review board approved retrospective study; patients with large geometry change (external body contour change >1 cm between planning CT and CBCT) were excluded. All patients received step‐and‐shoot IMRT or VMAT treatments in our clinic for four anatomic sites: HN, lung, pancreas, and prostate. If the patient received IMRT (or VMAT) treatment, a complementary VMAT (or IMRT) plan was retrospectively made, with dose distributions comparable to the original clinical plan. In this way, a total of 80 plans were included in this study.

All patients were treated on Varian linacs (iX, Trilogy, or TrueBeam; Varian Medical Systems, Palo Alto, CA, USA). Each linac was integrated with an onboard kV CBCT (OBI; Varian). All patients underwent CBCT scans prior to their first treatment. CBCTs of the first treatment day were used for subsequent dose calculation to minimize potential anatomical variations between CT simulation and the start of the radiation treatment regimen. The time interval between CT simulation and the CBCT is 13.3 ± 4.3 days. Patient selection intentionally mimicked the real clinical world, so that CBCT images with artifacts or relatively low image quality were still included in the study.

### 2.B Patient‐specific stepwise HU‐to‐density curve

**Figure 1:**
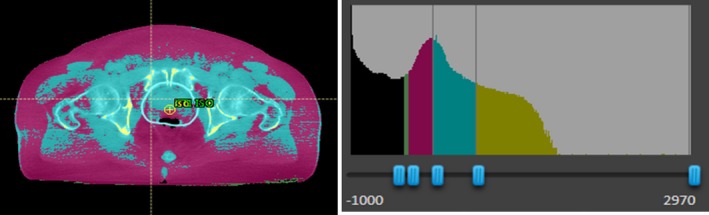
Example showing mass density on CBCT images (left) and HU thresholds to define material type (right). Mass density was assigned to each voxel via mapping CBCT HUs to six classes of materials (patient‐specific HU‐to‐density table; right). HU threshold to define different materials can be adjusted via best match with known tissue on CBCT. Black = air; pink = adipose; light blue = tissue; gold = cartilage/bone (lung and other material not shown).

Unlike the CT‐based planning that uses only a single CT‐to‐electron density calibration curve in the treatment planning system, a patient‐specific stepwise HU‐to‐density curve was created for each patient, assigning each voxel of CBCT images to one of the following categories of material (mass density): air (0.00121 g/cm^3^), lung (0.26 g/cm^3^), adipose (0.95 g/cm^3^), tissue (1.05 g/cm^3^), cartilage/bone (1.6 g/cm^3^), and other (3 g/cm^3^). The treatment planning system is RayStation V5.0 (RaySearch Laboratories; Stockholm, Sweden), which provides the tool to adjust the HU threshold for each material via best match with the known material type (Fig. 1). The HU threshold was adjusted for each patient. The optimal thresholds were attained by identifying the range of HUs for each material category based on the CBCT. The corresponding mass densities were used in dose calculation.

### 2.C CBCT‐based dose calculation accuracy

**Figure 2:**
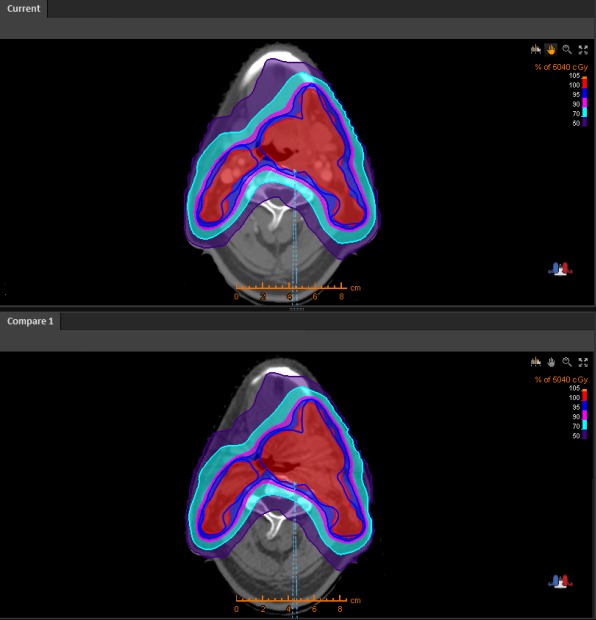
Planning CT (top) and CBCT (bottom) for a patient with head and neck cancer. Blue lines indicate PTV, and the color wash indicates the percentage of the prescription dose.

For each patient, CBCT scans were transferred to the RayStation treatment planning system and then registered to the planning CT based on the bony anatomy. Contours, such as planning target volume (PTV) and OARs, were copied from the planning CT to the CBCT image via rigid registration. The dose was recalculated using the original dose calculation algorithm, but using the CBCT image for both IMRT and VMAT plans. The dose calculated based on CBCT was compared to that based on planning CT (Fig. 2). Differences between the two plans were documented for the following dose–volume variables: dose received by 98% of the PTV (D98%, near‐minimum dose), PTV D95%, PTV D2% (near‐maximum dose), PTV mean dose, and PTV volume receiving ≥95% of prescription dose (V95%). Gamma analysis was performed using a criterion of 3%/3 mm for three dose zones: the zone receiving ≥90% of maximum dose (high‐dose region), the zone receiving 70%–90% of maximum dose (medium‐dose region), and the zone receiving 20%–70% of maximum dose (low‐dose region). These three zones represented three types of regions of interest: PTV, OARs adjacent to PTV, and OARs/normal tissue at some distance from the PTV and receiving low dose.

### 2.D Comparison between CT and CBCT‐based replanned dosimetry

Retrospective data from 12 additional patients with head and neck cancer were selected for evaluation of the method. These 12 patients were not included in the original cohort of 40 patients described above. Because of weight loss and source‐to‐skin dose changes, these patients had been rescanned based on clinicians’ judgments and evaluated based on the new CT results. CBCT images acquired within 3 days of the rescanned CT images were available for these patients. The original IMRT/VMAT plans were calculated based on the rescanned CT and CBCT images. As a result of weight loss, the largest change among the investigated variables was PTV D2% (near‐maximum dose). The other variables listed above had small but better changes, i.e., the PTV coverage was better, so only changes in PTV D2% between original plans and rescanned CT/CBCT images were compared. The Pearson linear correlation coefficient was calculated.

## 3 RESULTS

### 3.A CBCT‐based dose calculation accuracy

Dose differences between planning CT‐based plans and CBCT‐based plans for PTV are summarized in Table 1. Gamma analysis results are shown in Table 2. CBCT‐based dose calculation accuracy does not correlate with the planning technique (VMAT vs IMRT). No difference was observed between VMAT and IMRT plans for any disease sites. Results for all disease sites are summarized in Tables 1 and 2.

**Table 1:** PTV dose–volume difference between CBCT‐based and CT‐based plans

Disease site	Head and neck (mean ± SD)	Lung (mean ± SD)	Pancreas (mean ± SD)	Pelvis (mean ± SD)	All patients (mean ± SD)
Mean dose difference	0% ± 0.6%	−0.4% ± 1.1%	−0.2% ± 1.0%	0.2% ± 1.3%	−0.1% ± 1.1%
D2% difference	−0.5% ± 0.6%	−0.9% ± 1.2%	−1.1% ± 1.1%	−0.4% ± 1.3%	−0.7% ± 1.1%
D95% difference	0.4% ± 0.7%	−0.2% ± 1.4%	0.0% ± 0.9%	0.4% ± 1.2%	0.2% ± 1.1%
D98% difference	0.6% ± 0.8%	−0.2% ± 1.2%	0.0% ± 0.9%	0.4% ± 1.1%	0.2% ± 1.0%
V95% difference	0.4% ± 0.4%	0.1% ± 0.7%	0.0% ± 0.5%	0.6% ± 0.9%	0.3% ± 0.8%

**Table 2:** Gamma analysis results (passing rate using 3%/3 mm criterion) comparing CBCT‐based plan dose and CT‐based planning dose

Disease site	Head and neck (mean ± SD)	Lung (mean ± SD)	Pancreas (mean ± SD)	Pelvis (mean ± SD)	All patients (mean ± SD)
High‐dose zones	98.3% ± 1.5%	96.1% ± 5.0%	99.1% ± 2.4%	100% ± 0%	99.0% ± 1.9%
Medium‐dose zones	92.9% ± 5.5%	98.7% ± 3.4%	100% ± 0%	98.9% ± 2.5%	97.6% ± 4.4%
Low‐dose zones	92.1% ± 7.2%	98.7% ± 2.2%	96.9% ± 4.9%	95.8% ± 5.7%	95.3% ± 6.0%

### 3.B Feasibility for evaluating dosimetry

The International Commission on Radiation Units & Measurements (ICRU) Report 83^22^ recommends that 85% of target points should meet the criteria of absorbed dose difference within 5% if points are located at low‐gradient areas (dose change <20%/cm) or that distance‐to‐agreement should be within 5 mm if points are located at high‐gradient areas (dose change >20%/cm). CBCT‐based dose accuracy was determined to be above the ICRU recommendation; therefore, the PTV dose–volume parameters (mean dose, D2%, D95%, D98%, and V95%) were used to decide the replanning time. Gamma analysis using the criterion of 3%/3 mm can serve the same purpose.

### 3.C Comparison between CT and CBCT‐based replanned dosimetry

**Figure 3:**
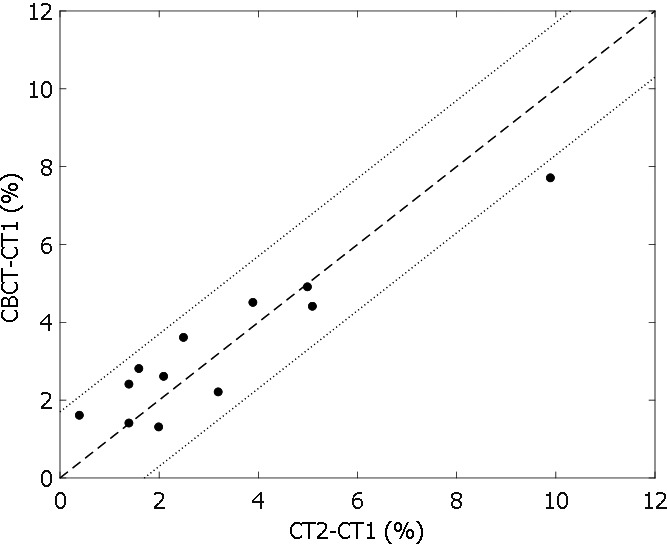
Change in PTV D2% relative to the prescription dose based on CBCT vs change based on CT2 for 12 patients with head and neck cancer who were rescanned because of weight loss.

Changes in near‐maximum dose of PTV (D2%) between initial planning CT and rescanned CT/CBCT were calculated for 12 patients with HN cancer. Changes based on CBCT and those based on rescanned CT are plotted in Fig. 3 and are significantly correlated (Pearson linear correlation coefficient = 0.93; *P* < 0.001). The change in D2% based on CBCT predicts the change based on rescanned CT, which was assumed to be ground truth. If 3% of the change in D2%, for example, was used as the replanning criterion, seven of the 12 patients could have avoided replanning procedures. Note that 3% of the change is an arbitrarily chosen criterion. The replanning criteria should depend on the clinical need, and it is beyond the scope of this paper.

## 4 DISCUSSION

### 4.A CBCT‐based dose calculation accuracy

Although many researchers^7^, ^8^, ^9^, ^10^, ^11^, ^12^, ^13^ have investigated CBCT‐based dose calculations and their use in replanning or online adaptive planning, clinical implementation has remained challenging because of the complexity of the technique or inability to achieve the accuracy required by treatment planning. In this study, we assessed the accuracy of the CBCT‐based dose calculations using patient‐specific stepwise HU‐to‐density curves and investigated the feasibility of using this method to determine replanning time. Six types of materials were used to convert the HU to density. A similar method (manually “overriding” density of all structures of interest on CBCT images) was investigated by Fotina et al.,^12^ who documented this as an attractive approach. Both their and our studies included pelvis patients and HN patients treated with IMRT. For their study, the dose or coverage differences (D2%, D98%, and V95%) between planning CT and CBCT were −3.2% ± 3.4%, 0.6% ± 1.8%, and 0.9% ± 2.9% for pelvis patients, and −2.3% ± 7.5%, 0.9% ± 7.1%, and −1.9% ± 1.4% for HN patients. Our method shows a slightly better dose accuracy: the dose or coverage differences (D2%, D98%, and V95%) between planning CT and CBCT were −0.4% ± 1.3%, 0.4% ± 1.1%, and 0.6% ± 0.9% for pelvis patients, and −0.5% ± 0.6%, 0.4% ± 0.7%, and 0.4% ± 0.4% for HN patients. Because most of treatment planning systems provide the function of density overriding, our method can be easily implemented in clinical practice. The CBCT dose was compared to the dose in the initial plan, which was assumed to be ground truth. Both delivery modalities (VMAT and IMRT) were compared, and we conclude that the accuracy of CBCT‐based dose calculation is not dependent on delivery technique. Four treatment sites (head and neck, lung, pancreas, and pelvis) were included in this study, and the accuracy of CBCT‐based dose calculation was slightly related to the treatment sites. Both dose statistics and gamma analysis showed that an accuracy of 3% is achievable for CBCT‐based dose calculations. The gamma analysis was performed for three dose zones representing PTV, OARs receiving high dose, and OARs receiving low dose.

### 4.B Feasibility to determine the best replanning time

The recently published ICRU Report 83 suggests defining dose accuracy with dose–volume statistics rather than the point dose, as recommended by ICRU Report 50 and 62; therefore, PTV dose–volume statistics (D2%, D95%, D98%, V95%) and gamma analysis were used to assess CBCT‐based dose accuracy in this study. Our results showed that CBCT‐based dose accuracy was much better than absorbed dose accuracy as suggested by ICRU Report 83; therefore, all of these parameters in this study are feasible for indication of the dose difference between initial planning dose and CBCT‐based dose. CBCT‐based dose can quantitatively provide the dosimetry change (at a 3% accuracy level) to the physician. Data from the 12 patients with HN cancer assessed for validation purposes showed that the change of PTV D2% was significantly correlated between CBCT data and rescanned CT data. With the 3% criterion, seven of these 12 patients could have avoided rescanning procedures, despite changes in weight and large changes in skin‐to‐source doses after initial planning. Changes in these parameters during treatment are feasible to help physicians decide whether a patient needs rescanning. Replanning criteria should be determined based on clinical need and rationale. Further investigation of guidelines for these criteria in specific clinical situations is needed before this method can be applied in the clinical setting. Specific OAR doses may be used to determine the time of replanning, depending on the case. Because CBCT is becoming a routine tool in imaging‐guided radiotherapy, the CBCT‐based dose calculation can be thought of as an extra benefit for patients, conveying the potential to avoid unnecessary rescanning, with resulting benefits in lower cumulative radiation dose, less treatment delay, and reduced medical costs.

### 4.C Limitation of the study

Although 40 patients were selected to minimize geometric differences between planning CT and CBCT, small differences may still exist. The dosimetry difference between the CBCT plan and planning CT mainly results from the patient‐specific stepwise HU‐to‐density curve, but geometric change, leading to dosimetric differences, cannot be excluded. However, without geometric change, dosimetric agreement between the two dose calculations would be expected to improve in this study.

This work investigates the feasibility of performing accurate dose calculations on CBCT images. We consider this calculation a necessary step toward implementing adaptive planning in our clinic. We do not address differences in anatomy observed between planning CT and CBCT as this involves clinical decision making. Actual implementation of CBCT‐based replanning into the routine clinical workflow is beyond the scope of this paper.

## 5 CONCLUSIONS

CBCT‐based dose calculations produced accuracy comparable to that of simulation CT. CBCT‐based dosimetry can guide the decision to replan during the course of treatment.

## CONFLICT OF INTEREST

The authors declare no conflict of interest.
